# Sleep disturbance and internalizing symptoms in adolescents: a moderated mediation model of self-control and mindfulness

**DOI:** 10.1186/s12888-024-05750-y

**Published:** 2024-04-24

**Authors:** Haoxian Ye, Nan Jiang, Sisi He, Fang Fan

**Affiliations:** Centre for Studies of Psychological Applications, Guangdong Key Laboratory of Mental Health and Cognitive Science, Ministry of Education Key Laboratory of Brain Cognition and Educational Science, School of Psychology, South China Normal University, Shipai Road, 510631 Guangzhou, China

**Keywords:** Sleep disturbance, Internalizing symptoms, Self-control, Mindfulness, Adolescents.

## Abstract

**Objectives:**

Despite accumulating evidence regarding the impact of sleep disturbance on internalizing symptoms among adolescents, the underlying psychological mechanisms remain inadequately explored. This study aimed to investigate a conceptual framework elucidating how sleep disturbance influences internalizing symptoms in adolescents through the mediating role of self-control, with mindfulness as a moderator.

**Methods:**

In this cross-sectional study, 1876 Chinese adolescents (Mage = 14.88 years, SD = 1.47 years, range = 12–19 years, 44.7% boys) completed the Youth Self-Rating Insomnia Scale (YSIS), Patient Health Questionnaire-9 (PHQ-9), Generalized Anxiety Disorder-7 (GAD-7), Brief Self-control Scale (BSCS), and Mindful Attention Awareness Scale-Children (MAAS-C) to provide data on sleep-related variables, internalizing symptoms (anxiety and depression), self-control, and mindfulness, respectively. The PROCESS macro for SPSS was applied to perform moderated mediation analysis.

**Results:**

Sleep disturbance demonstrated a significant positive correlation with internalizing symptoms in adolescents, including anxiety (β = 0.481, *p* < 0.001) and depression (β = 0.543, *p* < 0.001). Self-control served as a mediator between sleep disturbance and two forms of internalizing symptoms. Moreover, mindfulness moderated the pathways from self-control to internalizing symptoms (for anxiety symptoms: β = 0.007, *p* < 0.001; for depression symptoms: β = 0.006, *p* < 0.001), and the mediating relationships were weaker for adolescents exhibiting higher levels of mindfulness.

**Conclusions:**

Our findings enhance understanding of the impact, pathways, and influencing factors of sleep disturbance on adolescent internalizing symptoms, suggesting the importance of enhancing mindfulness levels in addressing self-control deficits and subsequently reducing internalizing symptoms among adolescents.

**Supplementary Information:**

The online version contains supplementary material available at 10.1186/s12888-024-05750-y.

## Introduction

Adolescence is a critical period for mental health, characterized by profound physical and psychological changes [1]. These changes heighten vulnerability to internalizing symptoms [2], such as depression and anxiety [3]. Recent meta-analyses indicate that a significant proportion of Chinese youth face these challenges, with about 19.85% struggling with depression [4] and 25% facing anxiety [5], particularly during challenging times like the COVID-19 pandemic. Given that internalizing symptoms could further lead to severe outcomes like social and emotional dysfunction, diminished academic performance, substance use, and suicidal ideation [6, 7], it is crucial to identify risk factors for these symptoms during adolescence.

One pivotal risk factor for internalizing symptoms is sleep disturbance [8], which emerges as a warning sign for internalizing symptoms and has a profound impact on these conditions [9]. Sleep disturbance could lead to deficits in emotion regulation, coping [10], and stress resistance [11], which have all been associated with subsequent internalizing problems [12]. While the impact of sleep disturbance on internalizing symptoms is increasingly acknowledged [13, 14], existing research predominantly explores biological mediators in these relationships, such as inflammatory markers [15], brain function [16], and grey matter structure [17]. This leaves a notable gap in understanding the mediating role that psychological and social factors play in these relationships. However, considering the relationships between sleep disturbance and adolescent internalizing symptoms are influenced by “bio-psycho-social” factors [18], it is necessary to explore the plausible psychological factors that mediate the relationship between sleep disturbance and internalizing symptoms. Doing so could unveil innovative approaches for the development of prevention and intervention strategies aimed at adolescents.

Self-control, a fundamental cognitive ability to regulate our thoughts and behaviors, may be a psychological mediator in the nexus between sleep disturbance and internalizing symptoms. Based on the strength model of self-control [19], this cognitive capability relies on a limited energy reserve, and energy depletion could lead to reduced self-control [20]. Moreover, this reserve is linked to brain glucose levels [21], while sleep plays a vital role in replenishing energy by restoring optimal glucose levels and other vital nutrients [22]. Thus, sleep disturbance may cause significant reductions in glucose levels, which could lead to emotional exhaustion [23], thereby impairing self-control [24, 25] and increasing susceptibility to internalizing symptoms [26]. This theoretical model is also supported by cross-sectional [27, 28] and longitudinal research [29], which emphasizes the key predictive role of low self-control in the onset of internalizing problems among adolescents. Taken together, the impact of sleep disturbance on adolescents’ internalizing symptoms may be mediated by reductions in self-control.

As the second peak of physiological development, adolescence often experiences significant imbalances in physical and mental development [30]. This imbalance challenges adolescents’ ability to maintain stable levels of self-control, making them vulnerable to fluctuations and exacerbating the mediating effect of impaired self-control in the relationships between sleep disturbance and internalizing symptoms. Thus, identifying protective factors that can mitigate the negative effects of low self-control on adolescent health becomes significant. Mindfulness is likely a psychological factor capable of buffering the risks associated with low self-control [31]. In line with the mindfulness-based stress reduction (MBSR) framework, mindfulness - a nonjudgmental awareness of experiences in the present moment - can enhance self-regulation by improving emotional and attentional control [32], buffering against the risk of low self-control [33]. In fact, the key role of mindfulness in counteracting self-control depletion has been supported by empirical studies [34]. Additionally, the feasibility and accessibility of mindfulness training make it a practical option for adolescents to enhance protective psychological resources [35, 36]. For example, brief mindfulness interventions, such as four 20-minute breathing meditation sessions, have demonstrated a rapid enhancement of present-moment awareness and overall mindfulness levels [37, 38]. Taken together, mindfulness is promising to be an efficient protective factor for adolescents to compensate for low self-control, helping alleviate the link between sleep disturbance, self-control, and internalizing symptoms.

**Fig. 1:**
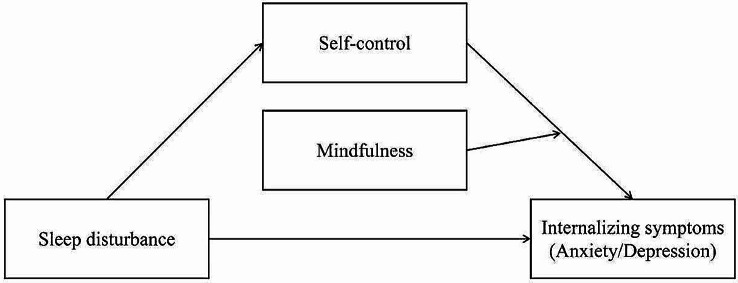
The hypothesized moderated mediation model

To the best of our knowledge, despite extensive research on the relationship between sleep disturbance and internalizing symptoms in adolescence [39], the psychological mechanisms underlying this link remain underexplored, particularly regarding the combined roles of self-control and mindfulness. Our integrated model seeks to fill this gap by offering a comprehensive framework that: (1) elucidates the direct impact of sleep disturbance on internalizing symptoms; (2) identifies self-control as a mediator; and (3) positions mindfulness as a crucial moderator within this dynamic (see Fig. 1). By elucidating the roles of self-control and mindfulness, this proposed framework provides a foundation for developing targeted interventions aimed at enhancing these psychological resources, thereby mitigating the adverse effects of sleep disturbance on adolescent mental health. Such insights are critical for informing evidence-based practices and contribute to the broader biopsychosocial understanding of sleep and mental health [18].

## Methods

### Participants

Convenience sampling was used to collect a large sample of students from 3 schools (1 primary school (grades 1–6), 1 junior high school (grades 7–9), and 1 secondary school (grades 7–12)) in one district of an eastern city in Guangdong, China. The age ranges of primary school, junior high school, and secondary school are 9–14, 11–16, and 11–19, respectively. We did not include grades 1–4 with the concern that they may not well understand the questionnaire due to their young age, and we also excluded students in grades 9 and 12 since they were preparing for the high school and college entrance exams, respectively. Finally, from April 7th to April 31st, 2023, a total of 2,229 students in grades 5–11 were invited to take part in the investigation.

As a means of controlling the quality of the survey responses, our exclusion standards were: (1) incorrect identity information; (2) an unrealistically short response time (below 5 min); and/or (3) inconsistent survey contents. These criteria were in line with the previous studies [39–41]. Therefore, a small number of participants met these exclusion criteria, leaving 1,876 valid responses.

### Procedures

This study was carried out in accordance with the Helsinki Declaration as revised in 1989 and approved by the Ethics Committees of the University of the corresponding author (SCNU-PSY-2023-191). Before the survey, the local education bureau and the school jointly sent out invitation letters for the survey to all students and their guardians. Students and their guardians are required to scan the Quick Response (QR) code or click the link of the questionnaire through mobile phones or computers to complete the web-based survey and read the electronic informed consent at first. If both the students and their guardians agreed to participate in the study, they could continue to complete the survey. Responses to the survey were kept confidential, and all students and guardians provided electronic informed consent with the right to withdraw freely during the test period. As the online platform needed to finish all items before submission, there was no missing data for the variables studied in the current research.

### Measurements

#### Sociodemographic information

In line with previous studies [43, 44], self-reporting techniques were used to gather common sociodemographic variables, including age, gender, ethnicity, single child status, parental married status, family incomes, father’s educational level, mother’s educational level, father’s job stability, mother’s job stability, immigrants’ generation (immigrated to the current residential city from other places), left-behind status (parents migrated to another city or region for work over 6 months), local resident, degree of impact by COVID-19 on life, lifestyles (smoking, alcohol intake, and exercise), self history of psychiatric illness, family history of psychiatric illness, and chronic physical illness.

#### Sleep disturbance

Four items extracted from the Youth Self-Rating Insomnia Scale (YSIS) [45] were used to assess sleep disturbance in the past month. These items focused on various aspects of sleep, including three common insomnia symptoms (difficulty initiating sleep (DIS), difficulty maintaining sleep (DMS), and early morning awakening (EMA)) and subjective sleep quality. The selection of these items aligned with the core features of sleep disturbance [46] and maintained consistency with prior research [41, 47, 48]. Responses to each item were recorded on a 5-point Likert scale. The items of DIS, DMS, and EMA ranged from 1- never, 2- <1 time/week, 3- 1-2 times/week, 4- 3-5 times/week, to 5- 6-7 times/week, while the item of subjective sleep quality ranged from 1- very good, 2- good, 3- fair, 4- poor, to 5- very poor [41, 49]. Scores from the four items were summed to generate a total score for the severity of sleep disturbance [41, 48]. In this study, the Cronbach’s alpha for these four items was 0.775.

#### Anxiety symptoms

The severity of anxiety symptoms experienced over the past two weeks was evaluated by the Chinese version of the GAD-7 [50], which exhibited strong psychometric properties in Chinese adolescents [51]. The ratings for each item range on a 4-point scale from 0 (not at all) to 3 (nearly every day). Item scores were added up to generate a total score, with a higher total score indicating a higher level of anxiety symptoms. In this study, its Cronbach’s alpha was 0.953.

#### Depression symptoms

The severity of depressive symptoms experienced over the past two weeks was assessed using the Chinese version of the 9-item Patient Health Questionnaire (PHQ-9) [52], which exhibited strong psychometric properties in Chinese adolescents [53]. The ratings for each item range on a 4-point scale from 0 (not at all) to 3 (nearly every day), and all the items were added together to get a total score. A higher total score denotes more severe depressive symptoms. In this study, its Cronbach’s alpha was 0.921.

#### Self-control

Self-control was evaluated by the Chinese version of the brief self-control scale (BSCS) [54], which had satisfactory-to-excellent psychometric properties among Chinese adolescents [55]. The BSCS included two domains: impulse control and self-discipline. Each item was rated on a 5-point Likert scale from 1 (strongly disagree) to 5 (strongly agree). All items were added together to get a total score, with a higher score indicating a higher level of self-control. In this study, its Cronbach’s alpha was 0.791.

### Mindfulness

Mindfulness was measured by the Chinese version of the Mindful Attention Awareness Scale-Children (MAAS-C) [56], which exhibits satisfactory psychometric properties among Chinese adolescents [57]. The MAAS-C is a 15-item scale developed to assess mindfulness in terms of trait, which is adapted from the Mindful Attention Awareness Scale (MAAS) [58]. Participants responded on a 6-point Likert scale ranging from 1 (always) to 6 (never), with higher scores indicating greater levels of mindfulness. In this study, its Cronbach’s α was 0.947.

### Data analyses

First, descriptive statistics were calculated for sociodemographic information, while Pearson correlations were adopted to examine the relationships among sleep disturbance, anxiety symptoms, depression symptoms, self-control, and mindfulness. Second, the PROCESS macro in SPSS was used to test for the mediation and moderating effects, with bootstrapping for parameter estimation and a sample size of 5000. Significant parameters were identified as those with 95% confidence intervals that did not include 0. To be specific, we first utilized Model 4 in PROCESS to test the mediating model, with sleep disturbance as an independent variable, self-control as a mediating variable, and depression symptoms and anxiety symptoms as dependent variables, respectively. Then, we utilized Model 14 in PROCESS to test the moderated mediation model, with sleep disturbance as a predictor, self-control as a mediator, mindfulness as a moderator, and anxiety symptoms and depression symptoms as outcomes, respectively. Sociodemographic variables were all included as covariates in the above analyses.

## Results

### Descriptive statistics of sample characteristics

A total of 1,876 participants were included in the analysis of the present study. Their mean (SD) age was 14.88 (1.47) years, ranging from 12 to 19. Boys made up 44.7% of this sample (*n* = 838), and the proportions for junior high school students and senior high school students were 60.3% (*n* = 1,131) and 39.7% (*n* = 727), respectively. Detailed sample characteristics are illustrated in Table 1.

### Assessment of common method variance

As self-reporting was the only approach used to collect data in the present study, common method variance may exist. To examine it, we conducted Harman’s one-factor test [58]. The results indicated that the first principal factor explained 38.58% of the variance (< 40%), suggesting that common method variance is not a significant concern in this study.

### Correlation analyses

Descriptive information and Pearson correlations for the studied variables are shown in Table 2. Sleep disturbance was significantly and positively associated with both anxiety symptoms and depression symptoms. Self-control was negatively correlated with sleep disturbance, anxiety symptoms, and depression symptoms. In addition, mindfulness was positively correlated with self-control and negatively correlated with sleep disturbance, anxiety symptoms, and depression symptoms.

### Mediation analyses

As shown in Tables 3 and 4, the findings demonstrated that sleep disturbance had a significant total effect on anxiety symptoms (β = 0.481, *p* < 0.001) and depression symptoms (β = 0.543, *p* < 0.001), respectively. Sleep disturbance was negatively associated with self-control (β = -0.316, *p* < 0.001), and self-control negatively predicted anxiety symptoms (β = -0.288, *p* < 0.001) and depression symptoms (β = -0.306, *p* < 0.001), respectively. The bootstrap results with 5000 re-samples revealed a significant indirect effect. Specifically, self-control played a mediating role in anxiety symptoms (effect = 0.147, BootSE = 0.016) and depression symptoms (effect = 0.170, BootSE = 0.017). The mediating effect accounted for 18.9% and 17.9% of the total effect of sleep disturbance on anxiety symptoms and depression symptoms, respectively.

### Moderated mediation analyses

**Fig. 2:**
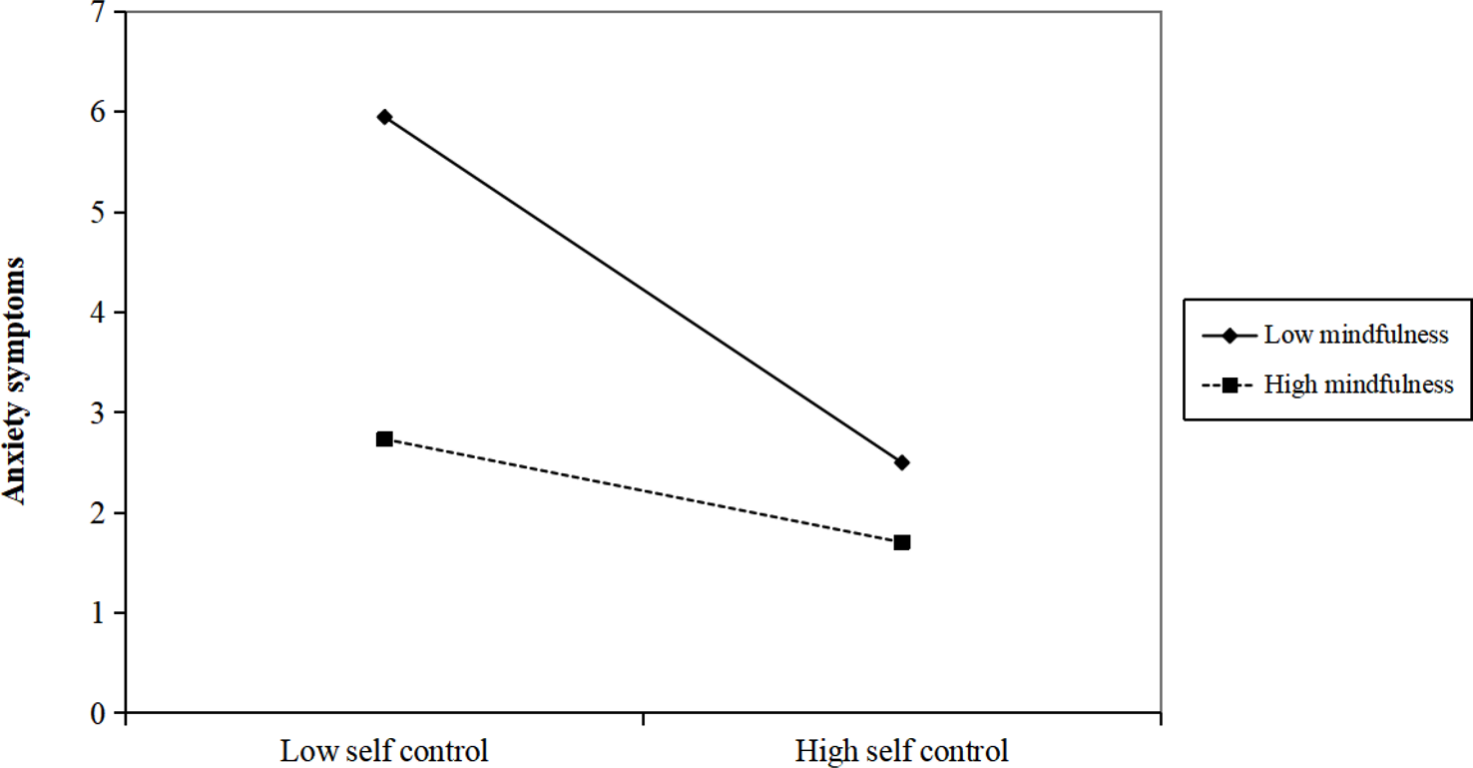
The interaction effect of self-control and mindfulness on anxiety symptoms

**Fig. 3:**
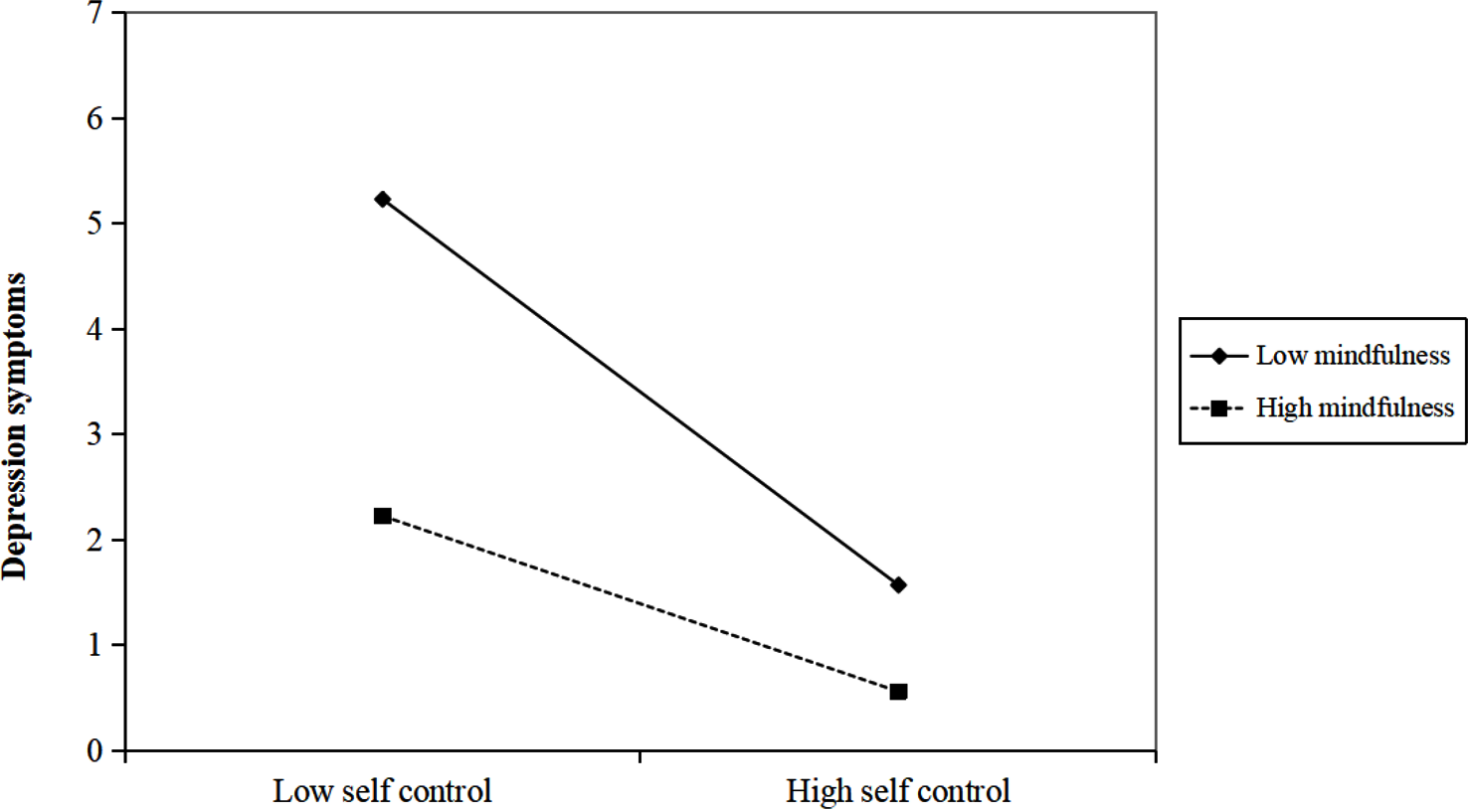
The interaction effect of self-control and mindfulness on depression symptoms

After controlling the influence of sociodemographic information, the interaction between mindfulness and self-control had a significant predictive effect on anxiety symptoms (β = 0.007, *p* < 0.001) and depression symptoms (β = 0.006, *p* < 0.001), respectively (see Tables 5 and 6). Based on the plots depicting that the relation between self-control and anxiety symptoms/depression symptoms separately for low and high levels of mindfulness (i.e., 1 SD below the mean and 1 SD above the mean, respectively) (see Figs. 2 and 3), our simple slope test showed, for adolescents with low levels of mindfulness, self-control have a significant negative predictive effect on anxiety symptoms (β_simple_ = -0.334, *p* < 0.001) and depression symptoms (β_simple_ = -0.366, *p* < 0.001); Yet, for those with high levels of mindfulness, self-control have a significant negative but weaker predictive effect on anxiety symptoms (β_simple_ = -0.105, *p* < 0.001) and depression symptoms (β_simple_ = -0.168, *p* < 0.001). In addition, as illustrated in Tables 5 and 6, the conditional indirect effect analysis showed that under high levels of mindfulness, the mediating effect of self-control in the relationship between sleep disturbance and internalizing symptoms was much weaker than under low levels of mindfulness.

### Sensitivity analyses

In line with the prior research using a moderated mediation model [60], we conducted a sensitivity analysis to ensure that the above findings were not influenced by potential confounders such as sociodemographic variables. To achieve this, we replicated all analyses without controlling for sociodemographic variables. As shown in Supplementary Materials (Tables S1-S4), there were no meaningful changes in the significance or magnitude of the findings from mediation analyses and moderated mediation analyses, indicating the robustness of our findings.

## Discussion

Through the examination of the moderated mediation model, this study elucidates the pathway through which sleep disturbance impacts internalizing symptoms by influencing self-control and identifies the conditions under which the interplay among sleep disturbance, self-control, and internalizing symptoms is most pronounced in adolescents. The principal findings are as follows: (1) a positive correlation between sleep disturbance and internalizing symptoms was discerned; (2) self-control served as a mediator in the relationship between sleep disturbance and internalizing symptoms; and (3) mindfulness played a moderating role in the connection between self-control and internalizing symptoms, indicating that adolescents with high mindfulness exhibited diminished susceptibility to internalizing symptoms resulting from self-control.

Consistent with prior investigations [61–63], our study establishes a positive association between sleep disturbance and internalizing symptoms, supporting the notion that sleep disturbance is a precursor to internalizing symptoms [64]. In fact, over the past decades, sleep disturbance has emerged as an early and dependable indicator of vulnerability to anxiety and depression in adolescents [13, 65]. Hence, implementing interventions targeting sleep, including the modification of adverse sleep habits and addressing symptoms of insomnia, proves crucial in alleviating internalizing symptoms among adolescents.

Complementing current research on biological mediators [15–17], our study unveils a novel psychological mediator, elucidating how sleep disturbance might compromise self-control, consequently leading to internalizing symptoms. Drawing insights from established literature [66, 67] and the strength model of self-control [22, 68], self-control hinges on a finite energy reserve, and its exertion depletes this reserve. Effective sleep, a primary avenue for energy restoration, plays a pivotal role in sustaining self-control. Therefore, pronounced sleep disturbance in adolescents may impede the replenishment of resources from the preceding day [69], resulting in attenuated self-control [20]. Furthermore, in addition to the documented positive correlations between self-control and internalizing symptoms [70, 71], researchers have also revealed the impact of specific self-control components on internalizing symptoms [72]. For instance, deficits in impulse control often manifest as difficulties in regulating emotions and behaviors, heightening the challenge of disengaging from anxious emotions [73]. Simultaneously, individuals with low self-discipline may be more prone to succumbing to temptations, leading to a higher likelihood of developing addictions [74, 75] and, consequently, an increased risk of depressive symptoms [76]. Taken together, our findings underscore the potential of sleep disturbance to diminish self-control, thereby contributing to the severity of internalizing symptoms.

Building upon this mediating pathway, our study unveils the moderating influence of mindfulness in the connection between self-control and internalizing symptoms. Among adolescents exhibiting high levels of mindfulness, the impact of self-control on internalizing symptoms diminishes. This buffering effect indicates that enhanced mindfulness might furnish adolescents with supplementary resources, such as emotional intelligence and psychological capital, fortifying their mental well-being [77]. Consequently, it mitigates the detrimental repercussions of low self-control [78]. Hence, our findings underscore the potential of mindfulness as a compensatory strategy amid the inherent instability of self-control during adolescence. The implementation of mindfulness-based training for adolescents may be a beneficial approach to attenuate the mediating effect of self-control in the relationship between sleep disturbance and internalizing symptoms.

Several limitations of this study should be acknowledged. First, the cross-sectional nature of the research design underscores the need for caution in establishing causal relationships. Future investigations would benefit from employing longitudinal or experimental designs to more effectively elucidate the direction of causality. Second, reliance on self-reported measurement may introduce potential response bias and inaccuracies in recall. Subsequent research should bolster the robustness of findings by integrating data from diverse sources, including peers, parents, and teachers, to validate the reported outcomes. In addition, given that the levels of sleep disturbance were evaluated subjectively, future research could consider incorporating polysomnography (PSG) to objectively assess physical sleep indicators and including adolescents diagnosed with sleep disorders to examine the current findings. Third, despite the proven effectiveness of the measurements used in the study, they might not fully capture all dimensions of sleep disturbance, internalizing symptoms, self-control, and mindfulness. Future research could benefit from employing a broader array of tools and methodologies to capture these constructs’ complexity more fully, thereby providing a more nuanced understanding of the relationships between sleep disturbance, self-control, mindfulness, and internalizing symptoms among adolescents. Lastly, the current sample of adolescents was drawn from a singular district, necessitating circumspection when extrapolating the findings more broadly. Future research should consider applying the proposed model to diverse adolescent samples to assess the generalizability of the results and explore the potential influences of cultural contexts on the outcomes.

### Conclusions and implications

Despite these limitations, the current findings advance our comprehension of the nexus between sleep disturbance and internalizing symptoms in adolescents, offering valuable insights for prospective intervention strategies. We identified self-control as a mediating factor and mindfulness as a moderating influence, delineating how and under what conditions sleep disturbance relates to internalizing symptoms. Importantly, the beneficial role of mindfulness in mitigating the impact of low self-control on internalizing symptoms was emphasized. Thus, our main findings have significant implications for the development and refinement of interventions aimed at mitigating internalizing symptoms among adolescents. Specifically, given the mediating role of self-control, interventions could incorporate techniques to enhance self-control, such as goal setting, self-monitoring, and developing coping strategies, which may reduce the vulnerability to internalizing symptoms stemming from sleep disturbance. Furthermore, the moderating effect of mindfulness suggests that mindfulness-based interventions emerge as a particularly effective strategy to compensate for the inherent instability of self-control in adolescents. Incorporating mindfulness practices into programs for adolescents could help cultivate a non-judgmental awareness of the present moment, improve self-regulation, and potentially buffer against the adverse effects of sleep disturbance on mental health. Altogether, the above interventions could more effectively support adolescent mental health, fostering resilience against the development of internalizing symptoms.

**Table 1:** Descriptive statistics of the overall sample (*n* = 1876)

		n	%
Age ^a^ [year, mean(SD)]	14.88 (1.47)	-	-
Gender	Boys	838	44.7
	Girls	1038	55.3
Grade [age]	7th [13.42 (0.72)]	569	30.3
	8th [14.35 (0.73)]	562	30.0
	10th [15.95 (0.59)]	387	20.6
	11th [16.86 (0.60)]	358	19.1
Ethnicity	Han ^b^	1858	99.0
	Others	18	1.0
Parental martial status	Married	1754	93.5
	Not current married ^c^	122	6.5
Immigrants’ second generation	Yes	155	8.3
Single child status	Yes	131	7.0
Left-behind status ^d^	Yes	231	12.3
Father has a stable job	Yes	1593	84.9
Mother has a stable job	Yes	1382	73.7
Self history of psychiatric illness	Yes	9	0.5
Chronic physical illness ^e^	Yes	106	5.7
Family history of psychiatric illness	Yes	11	0.6
Local resident	Yes	1520	81.0
	No	356	19.0
Family incomes	< 5000	446	23.8
	5000–10,000	666	35.5
	10,000–20,000	174	9.3
	> 20,000	43	2.3
	Unknown	547	29.2
Father’s educational level	Illiteracy	280	14.9
	Elementary school	986	52.6
	Junior high school	398	21.2
	Senior high school	136	7.2
	Junior college	73	3.9
	College	3	0.2
Mother’s educational level	Illiteracy	480	25.6
	Elementary school	908	48.4
	Junior high school	357	19.0
	Senior high school	88	4.7
	Junior college	40	2.1
	College	3	0.2
Degree of impact by COVID-19 on life	No impact	485	25.9
	Minimal impact	660	35.2
	Moderate impact	554	29.5
	Significant impact	149	7.9
	Very significant impact	28	1.5
Alcohol intake	Never	1718	91.6
	Sometime	154	8.2
	Often	4	0.2
	Everyday	0	0.0
Exercise	Never	96	5.1
	Sometime	1187	63.3
	Often	438	23.3
	Everyday	155	8.3
Smoking	Never	1844	98.3
	Sometime	19	1.0
	Often	1	0.1
	Everyday	5	0.3
	Quit smoking	7	0.4

*Note*

^a^ The range of age in the current sample was 12–19

^b^ Han is the major ethnic group in China

^c^ Not current married included separated, divorced, and widowed

^d^ Live separately from one or both parents for more than 6 months

^e^ Chronic physical conditions referred to having at least one of arthritis angina, asthma, diabetes, visual impairment, or hearing problems

**Table 2:** Descriptive statistics of the studied variables

Variables	M	SD	1	2	3	4	5
1.Mindfulness	61.13	16.21	1				
2.Self-control	23.39	4.99	0.44^***^	1			
3.Depression symptoms	5.57	5.33	−0.47^***^	−0.53^***^	1		
4.Anxiety symptoms	3.98	4.91	−0.45^***^	−0.49^***^	0.87^***^	1	
5.Sleep disturbance	7.50	3.04	−0.39^***^	−0.39^***^	0.61^***^	0.54^***^	1

*Note*

^*^p < 0.05, ^**^p < 0.01, ^***^p < 0.001

**Table 3:** Results of the mediation analysis (depression symptoms as dependent variable)

Variables	Model 1 (depression symptoms)		Model 2 (self-control)		Model 3 (depression symptoms)	
	β	t	β	t	β	t
Age	0.063^***^	3.465	−0.103^***^	-4.813	-0.032	1.849
Gender	0.129^***^	7.041	−0.046^*^	-2.143	0.115^***^	6.710
Ethnicity	-0.027	-1.517	0.001	0.047	-0.027	-1.607
Single child status	-0.001	-0.023	−0.006	-0.257	-0.002	-0.123
Parental married status	0.012	0.646	0.012	0.518	0.016	0.891
Family incomes	0.017	0.936	−0.004	-0.209	0.015	0.992
Father’s educational level	-0.006	-0.300	0.007	0.294	-0.004	-0.208
Father’s job stability	0.004	0.233	−0.010	-0.449	0.001	0.076
Mother’s educational level	-0.019	-0.882	0.003	1.001	-0.011	-0.560
Mother’s job stability	0.002	0.099	0.001	0.012	0.002	0.111
Left-behind status	-0.040^*^	-2.097	0.063^**^	0.790	-0.021	-1.170
Immigrants’ second generation	0.021	1.147	−0.016	-0.734	0.016	0.946
Local resident	-0.000	-0.007	0.022	1.000	0.007	0.377
Degree of impact by COVID-19 on life	0.025	1.404	−0.023	-1.108	0.018	1.077
Family history of psychiatric illness	-0.029	-1.657	0.005	0.263	-0.028	-1.674
Self history of psychiatric illness	-0.047^**^	-2.670	0.048^*^	2.288	-0.033^*^	-1.976
Chronic physical illness	-0.047^**^	-2.654	0.031	1.521	-0.037^*^	-2.256
Exercise	-0.032	-1.728	0.129^***^	5.911	0.007	0.420
Smoking	0.031	1.665	−0.023	-1.082	0.023	1.367
Alcohol intake	0.146^***^	7.874	−0.132^***^	-6.072	0.106^***^	6.039
Sleep disturbance	0.543^***^	29.557	−0.316^***^	-14.659	0.446^***^	24.633
Self-control	-	-	-	-	-0.306^***^	-16.568
*R* ^*2*^	0.434		0.221		0.507	
*F*	67.61		25.09		86.54	

*Note*

^*^p < 0.05, ^**^p < 0.01, ^***^p < 0.001

**Table 4:** Results of the mediation analysis (anxiety symptoms as dependent variable)

Variables	Model 1 (anxiety symptoms)		Model 2 (self-control)		Model 3 (anxiety symptoms)	
	β	t	β	t	β	t
Age	0.064^**^	3.298	−0.103^***^	−4.813	0.035	1.861
Gender	0.128^***^	6.527	−0.046^*^	−2.143	0.115^***^	6.157
Ethnicity	−0.037	−1.953	0.001	0.047	−0.037^*^	−2.042
Single child status	−0.007	−0.346	−0.006	−0.257	−0.008	−0.450
Parental married status	0.003	0.160	0.012	0.518	0.007	0.341
Family incomes	0.002	0.114	−0.004	−0.209	0.001	0.051
Father’s educational level	0.011	0.511	0.007	0.294	0.014	0.636
Father’s job stability	−0.015	−0.773	−0.010	−0.449	−0.018	−0.964
Mother’s educational level	−0.031	−1.385	0.003	1.001	−0.024	−1.126
Mother’s job stability	0.003	0.181	0.001	0.012	0.004	0.195
Left-behind status	−0.045^*^	−2.216	0.063^**^	2.790	−0.027	−1.404
Immigrants’ second generation	0.024	1.234	−0.016	−0.734	0.019	1.056
Local resident	0.007	0.343	0.022	1.000	0.013	0.694
Degree of impact by COVID-19 on life	0.026	1.373	−0.023	−1.108	0.019	1.077
Family history of psychiatric illness	−0.040^*^	−2.092	0.005	0.263	−0.038^*^	−2.117
Self history of psychiatric illness	−0.041^*^	−2.144	0.048^*^	2.288	−0.027	−1.495
Chronic physical illness	−0.064^***^	−3.393	0.031	1.521	−0.055^**^	−3.068
Exercise	−0.009	−0.445	0.129^***^	5.911	0.028	1.485
Smoking	0.036	1.833	−0.023	−1.082	0.029	1.570
Alcohol intake	0.124^***^	6.264	−0.132^***^	−6.072	0.086^***^	4.534
Sleep disturbance	0.481^***^	24.515	−0.316^***^	−14.659	0.390^***^	19.833
Self-control	-	-	-	-	−0.288^***^	−14.333
*R* ^*2*^	0.353		0.221		0.417	
*F*	48.07		25.09		60.28	

*Note*

^*^p < 0.05, ^**^p < 0.01, ^***^p < 0.001

**Table 5:** Result of the moderated mediation analysis (depression symptoms as dependent variable)

Outcome variable: Self-control	β	t	SE	p
Constant	-0.064	-0.014	4.511	0.989
Sleep disturbance	-0.518	-14.659	0.035	< 0.001
*R* ^*2*^	0.221			
*F*	25.09			
***Outcome variable: Depression symptoms***	*β*	*t*	*SE*	*p*
Constant	2.395	0.642	3.731	0.521
Sleep disturbance	0.649	21.796	0.032	< 0.001
Self-control	-0.267	-13.236	0.020	< 0.001
Mindfulness	-0.061	-10.116	0.006	< 0.001
Self-control × Mindfulness	0.006	6.482	0.001	< 0.001
*R* ^*2*^	0.539			
*F*	90.25			
***Conditional indirect effect analysis at different values of mindfulness (M ± SD)***	*β*	*BootSE*	*BootLLCI*	*BootULCI*
*M−1SD*	0.190	0.024	0.144	0.236
*M*	0.138	0.016	0.107	0.172
*M + 1SD*	0.087	0.014	0.060	0.116

**Table 6:** Result of the moderated mediation analysis (anxiety symptoms as dependent variable)

Outcome variable: Self-control	β	t	SE	p
Constant	-0.064	-0.014	4.511	0.990
Sleep Disturbance	-0.518	-14.659	0.035	< 0.001
*R* ^*2*^	0.221			
*F*	25.09			
***Outcome variable: Anxiety symptoms***	*β*	*t*	*SE*	*p*
Constant	3.221	0.866	3.720	0.387
Sleep Disturbance	0.541	17.050	0.032	< 0.001
Self-control	-0.224	-11.161	0.020	< 0.001
Mindfulness	-0.061	-10.122	0.006	< 0.001
Self-control × Mindfulness	0.007	7.880	0.001	< 0.001
*R* ^*2*^	0.460			
*F*	65.73			
***Conditional indirect effect analysis at different values of mindfulness (M ± SD)***	*β*	*BootSE*	*BootLLCI*	*BootULCI*
*M−1SD*	0.178	0.023	0.134	0.224
*M*	0.116	0.015	0.088	0.147
*M + 1SD*	0.054	0.013	0.031	0.080

### Electronic supplementary material

Below is the link to the electronic supplementary material.

Supplementary Material 1

## Data Availability

The datasets used and/or analyzed during the current study are available from the corresponding author upon reasonable request.
