# Supplementary material for: Sleep disturbance and internalizing symptoms in adolescents: a moderated mediation model of self-control and mindfulness
Source: BMC Psychiatry. 2024 Apr 24;24:310. doi: 10.1186/s12888-024-05750-y (PMC11040859; doi:10.1186/s12888-024-05750-y)
Supplement: Supplementary file 1 — Supplementary Material 1 [file 12888_2024_5750_MOESM1_ESM.docx]

**Supplementary materials**

**Table S1** Results of the mediation analysis (depression symptoms as dependent variable).

| Variables | Model 1  (depression symptoms) | | Model 2  (self-control) | | Model 3  (depression symptoms) | |
| --- | --- | --- | --- | --- | --- | --- |
|  | *β* | *t* | *β* | *t* | *β* | *t* |
| Sleep disturbance | 0.610^***^ | 33.293 | -0.391^***^ | -18.378 | 0.473^***^ | 26.017 |
| Self-control | - | - | - | - | -0.349^***^ | -19.172 |
| *R^2^* | 0.372 | | 0.153 | | 0.475 | |
| *F* | 1108.41 | | 337.76 | | 846.39 | |

**Note:**

^*^*p* < 0.05, ^**^*p* < 0.01, ^***^*p* < 0.001.

**Table S2** Results of the mediation analysis (anxiety symptoms as dependent variable).

| Variables | Model 1  (anxiety symptoms) | | Model 2  (self-control) | | Model 3  (anxiety symptoms) | |
| --- | --- | --- | --- | --- | --- | --- |
|  | *β* | *t* | *β* | *t* | *β* | *t* |
| Sleep disturbance | 0.544^***^ | 28.092 | -0.391^***^ | -18.378 | 0.418^***^ | 21.219 |
| Self-control | - | - | - | - | -0.325^***^ | -16.497 |
| *R^2^* | 0.296 | | 0.153 | | 0.386 | |
| *F* | 789.13 | | 337.76 | | 587.73 | |

**Note:**

^*^*p* < 0.05, ^**^*p* < 0.01, ^***^*p* < 0.001.

**Table S3** Result of the moderated mediation analysis (depression symptoms as dependent variable).

| ***Outcome variable: Self-control*** | *β* | *t* | *SE* | *p* |
| --- | --- | --- | --- | --- |
| Constant | 4.810 | 17.034 | 0.282 | <0.001 |
| Sleep disturbance | -0.642 | -18.378 | 0.035 | <0.001 |
| *R^2^* | 0.153 |  |  |  |
| *F* | 337.76 |  |  |  |
| ***Outcome variable: Depression symptoms*** | *β* | *t* | *SE* | *p* |
| Constant | -0.181 | -0.710 | 0.256 | 0.480 |
| Sleep disturbance | 0.737 | 23.004 | 0.032 | <0.001 |
| Self-control | -0.308 | -15.384 | 0.020 | <0.001 |
| Mindfulness | -0.064 | -10.269 | 0.006 | <0.001 |
| Self-control × Mindfulness | 0.006 | 6.747 | 0.001 | <0.001 |
| *R^2^* | 0.511 |  |  |  |
| *F* | 487.78 |  |  |  |
| ***Conditional indirect effect analysis at different values of mindfulness (M ± SD)*** | *β* | *BootSE* | *BootLLCI* | *BootULCI* |
| *M - 1SD* | 0.264 | 0.028 | 0.208 | 0.319 |
| *M* | 0.197 | 0.019 | 0.159 | 0.235 |
| *M + 1SD* | 0.131 | 0.017 | 0.098 | 0.165 |

**Table S4** Result of the moderated mediation analysis (anxiety symptoms as dependent variable).

| ***Outcome variable: Self-control*** | *β* | *t* | *SE* | *p* |
| --- | --- | --- | --- | --- |
| Constant | 4.810 | 17.034 | 0.282 | <0.001 |
| Sleep Disturbance | -0.642 | -18.378 | 0.035 | <0.001 |
| *R^2^* | 0.153 |  |  |  |
| *F* | 337.76 |  |  |  |
| ***Outcome variable: Anxiety symptoms*** | *β* | *t* | *SE* | *p* |
| Constant | -0.640 | -2.526 | 0.254 | 0.012 |
| Sleep Disturbance | 0.581 | 18.272 | 0.032 | <0.001 |
| Self-control | -0.255 | -12.887 | 0.020 | <0.001 |
| Mindfulness | -0.063 | -10.299 | 0.006 | <0.001 |
| Self-control × Mindfulness | 0.007 | 8.099 | 0.001 | <0.001 |
| *R^2^* | 0.432 |  |  |  |
| *F* | 356.33 |  |  |  |
| ***Conditional indirect effect analysis at different values of mindfulness (M ± SD)*** | *β* | *BootSE* | *BootLLCI* | *BootULCI* |
| *M - 1SD* | 0.243 | 0.027 | 0.191 | 0.297 |
| *M* | 0.164 | 0.018 | 0.129 | 0.199 |
| *M + 1SD* | 0.084 | 0.015 | 0.055 | 0.114 |
